# Context-Aware Phylogenetic Trees for Phylogeny-Based Taxonomy Visualization

**DOI:** 10.3389/fgene.2022.891240

**Published:** 2022-05-18

**Authors:** Gizem Kaya, Chisom Ezekannagha, Dominik Heider, Georges Hattab

**Affiliations:** Department of Mathematics and Computer Science, University of Marburg, Marburg, Germany

**Keywords:** phylogeny, taxonomy, genomics, phylogeny-based taxonomy, visualization, phylogenetic tree, icicle

## Abstract

Sustained efforts in next-generation sequencing technologies are changing the field of taxonomy. The increase in the number of resolved genomes has made the traditional taxonomy of species antiquated. With phylogeny-based methods, taxonomies are being updated and refined. Although such methods bridge the gap between phylogeny and taxonomy, phylogeny-based taxonomy currently lacks interactive visualization approaches. Motivated by enriching and increasing the consistency of evolutionary and taxonomic studies alike, we propose Context-Aware Phylogenetic Trees (CAPT) as an interactive web tool to support users in exploration- and validation-based tasks. To complement phylogenetic information with phylogeny-based taxonomy, we offer linking two interactive visualizations which compose two simultaneous views: the phylogenetic tree view and the taxonomic icicle view. Thanks to its space-filling properties, the icicle visualization follows the intuition behind taxonomies where different hierarchical rankings with equal number of child elements can be represented with same-sized rectangular areas. In other words, it provides partitions of different sizes depending on the number of elements they contain. The icicle view integrates seven taxonomic rankings: domain, phylum, class, order, family, genus, and species. CAPT enriches the clades in the phylogenetic tree view with context from the genomic data and supports interactive techniques such as linking and brushing to highlight correspondence between the two views. Four different use cases, extracted from the Genome Taxonomy DataBase, were employed to create four scenarios using our approach. CAPT was successfully used to explore the phylogenetic trees as well as the taxonomic data by providing context and using the interaction techniques. This tool is essential to increase the accuracy of categorization of newly identified species and validate updated taxonomies. The source code and data are freely available at https://github.com/ghattab/CAPT.

## 1 Introduction

Defining and classifying organisms is a difficult task that biologists still face today. While naming entities is an important task, it is also essential to examine their similarities and differences in order to reach a consensus classification for a newly discovered entity or species. Considering that the classification of organisms has always been a very interesting task, mankind has been drawn to this subject since ancient times. From Aristotle, to Carolus Linnaeus, to Charles Darwin, the conceptual representation has evolved at the same pace as the understanding of species. The very first work by Aristotle classified animals and plants in ascending order (Aristotle, 1991). The Linnaean system employs a hierarchical form where the categorization of the species under different taxa was justified by comparing their morphological and physical traits (Schuh, 2003). In comparison, Darwin coined the term *Common Descent*, which describes the notion that the entire species descended from one common ancestor, and as a result, they are all related (Jenkin, 1867). The representation in the form of a tree with a common ancestor maintains its prominence in modern evolutionary biology, and this type of visualization is treated under the concept of a phylogenetic tree. Therefore, phylogenetic trees are now used to analyze the evolution of life, which Darwin had already examined with the metaphor of the *Tree of Life*. In modern phylogeny, the process of establishing relationships between organisms is becoming increasingly accurate thanks to advances in molecular analysis. Indeed, genomic sequences provide more reliable information than phenotypes.

In this respect, the taxonomic classification of species is retrieved from the available genomic sequences by applying various computational methods (Hugenholtz et al., 2016). This new era of taxonomy makes a specific visualization of phylogeny-based taxonomy necessary. Since the hierarchical structure between taxa is not time-dependent, it is not convenient to represent taxonomy by phylogenetic trees. To complement phylogenetic information with phylogeny-based taxonomy, we offer linking two interactive visualizations which compose two simultaneous views: the phylogenetic tree view and the taxonomic icicle view. These views comprise the interactive web tool for Context-Aware Phylogenetic Trees (CAPT) and support users in exploration- and validation-based tasks. The rationale behind such a structure is that the derived taxonomy is phylogeny-based, and there is a need to visually and interactively connect both views. To address this bi-fold problem, we shift the prevailing focus from the creation of phylogenetic trees to the visualization of phylogeny-based taxonomy (the icicle) and its interaction with a given phylogenetic tree.

Thanks to its space-filling properties, the proposed icicle visualization follows the intuition behind taxonomies where different hierarchical rankings with equal number of child elements can be represented with same-sized rectangular areas. In other words, it provides partitions of different sizes depending on the number of elements they contain. Compared to other visualization methods, this makes the icicle visualization more efficient than a node-link diagram and a tree-map visualization. Moreover, the icicle view integrates seven taxonomic rankings: domain, phylum, class, order, family, genus, and species. The CAPT web tool enriches the clades in the phylogenetic tree view with context from the genomic data and supports interactive techniques such as linking and brushing to highlight correspondence between the two views. Four different use cases were created by relying on the Genome Taxonomy DataBase (GTDB) to evaluate CAPT. They follow the scope of the GTDB and include the domain categories of Archaea and Bacteria.

A sensitivity analysis was carried out to evaluate the performance of CAPT. It consisted of calculating the average time needed to draw the icicle visualization given different numbers of selections excluding or including data preprocessing. On average, ten selected species on a phylogenetic tree required about 1.2 and 3.5 ms when excluding and including data preprocessing, respectively. CAPT was successfully used to explore the phylogenetic trees defined in the use cases as well as the associated taxonomic context. This tool is essential to increase the accuracy of categorization of newly identified species and validate updated taxonomies.

## 2 Related Work

The availability of large numbers of complete genomic sequences has influenced the historical practice of taxonomy. The use of morphology and physical characteristics to determine a taxonomic category of species has been repeatedly criticized because genes provide more reliable information than phenotypes for understanding evolutionary relationships. In this section, we present related work on the taxonomic computational methods currently used, taxonomy visualization methods, and hierarchical visualization methods.

### 2.1 Computational Methods for Taxonomy

The identification of a species category and assignment have long relied on morphological and physical traits. Thanks to the genomics era, it is possible today to establish a more reliable understanding of evolutionary relationships and obtain genome-derived or phylogeny-based taxonomies (Hugenholtz et al., 2016). Driven by the exploration of genetic diversity and the understanding of the species boundaries, it is important that computational methods achieve consistent outcomes to support both exploration- and validation-based tasks in taxonomic studies (Jain et al., 2018; Harrison & Larson, 2014). Two reasons motivate such an endeavor: obtaining a more coherent tree of life (Hugenholtz et al., 2016) and finding and validating with accuracy the taxonomic assignment of novel species (Godfray, 2002).

There are currently two main methods for the taxonomy of species: 16s rRNA gene sequencing and the Average Nucleotide Identity (ANI). The first method examines the relative position of a genome to other species by relying on the 16s rRNA gene (Godfray, 2002; Chaumeil et al., 2020; Konstantinos et al., 2017; Rodriguez-R et al., 2018). The 16s rRNA gene sequencing relies on clustering the resulting sequences based on their similarities. Operational Taxonomic Units or OTUs are then created and compared to existing ones in reference databases to assess likely assignments for taxonomy. Examined sequences are assessed by relying on the percentage of identity (>80% identity same phylum; >95% same genus; >97% highly related or the same species) (Parks et al., 2020; Schloss & Handelsman, 2005). Although widely used, this method relies on a single gene and is criticized for leading to low phylogenetic resolution at the species level (Segata et al., 2013). In other words, differences between closely related species cannot be detected using this method alone. The second method or ANI is generally preferred as it produces more accurate results and better defines species borders (Harrison & Larson, 2014; Godfray, 2002; Parks et al., 2020). It is made possible by computing the mean of all shared orthologs between two genomes (Godfray, 2002; Parks et al., 2020). An organism belongs to the same species if they have ≥95% ANI. Although preferred, it requires a comparison of whole genome sequences and entails intensive computations for their alignments. To facilitate the adoption of the ANI method, three main tools are known to increase the efficiency of calculations and provide coherent taxonomic categorization, namely the GTDB-Tk, PhyloPhlAn, and MiGA (Chaumeil et al*.*, 2020; Segata et al., 2013; Rodriguez-R et al., 2018).

Although current efforts at taxonomic categorization have made serious progress, it should be noted that phylogeny-based taxonomy relies on inferring phylogenies. Indeed, the use of multiple genes to infer species phylogenies is favored. Moreover, it is recognized that a gain in accuracy is sometimes accompanied by the reinforcement of certain systematic biases. This is particularly relevant when incorrect partitions and phylogenies have spuriously high bootstrap support (Gadagkar et al., 2005; Kubatko & Degnan, 2007).

### 2.2 Taxonomy Visualization

Due to the diversity of methods and approaches to alternative taxonomic classification, taxonomy is a highly controversial topic. In this context, visualization of taxonomy is essential to facilitate revision work, and as such, should provide both an overview and details of large datasets (Zhong et al., 1999; Wildpaner et al., 2001).

Most related work has preferred to present the result of taxonomy analysis using tree-based visualizations, including classification trees and node-link tree diagrams (Zhong et al., 1999; Parr et al., 2004; Rost & Bornberg-Bauer, 2002; Plaisant et al., 2002). In the particular case of phylogenetic trees, they were deemed incompatible with the representation of taxonomy for two reasons. First, in phylogenetic trees, only root and leaf nodes have taxonomic names and rankings. On the contrary, the internal nodes that indicate relationships between two neighboring species are not named and do not necessarily have a taxonomic rank (Zhong et al., 1999). The length of a branch represents the evolutionary distance, in turn leading to a variation in the branch length (Choi et al., 2000). Second, a parent node of any two child nodes does not necessarily belong to a higher taxonomic ranking.

Three major methods address taxonomy visualization: the HIerarchical CLAssification System or HICLAS was introduced to tackle the incompatibility of phylogenetic trees by adopting the logic of classification trees (Zhong et al., 1999), the taxonomy Workbench was introduced to visualize taxonomic trees as a node-link diagram or a tabulated list (Wildpaner et al., 2001), and SpaceTree was introduced as a tree browser to handle very large data sets (Plaisant et al., 2002). First, HICLAS adopted the representation of hierarchies with branches of equal length; where a parent node belongs to a higher classification level. Each node can be represented by a view of the taxon, including the taxon name, author, data, and corresponding publication (Zhong et al., 1996). Unlike a phylogenetic tree and thanks to a static number of taxonomic levels, the resulting classification tree has a fixed maximum number of total nodes (Zhong et al., 1999; Parr et al., 2004). TreeWiz is another noteworthy tool, capable of representing very large datasets as classification trees with interactive features such as zooming and filtering (Rost & Bornberg-Bauer, 2002). Second, the taxonomy Workbench reduces the visual complexity, it allows users to navigate a given view and show only the immediate environment of a selected taxon. As the number of nodes involved in creating the tree increases, the visualization becomes more complex. To alleviate this problem, Workbench integrated interactive features to work on a per-view basis. Third and last, SpaceTree builds on the conventional node-link tree diagrams and combines the classical node-link structure with zooming to expand and collapse part of a tree. Other interactive enhancements have been made by TaxonTree, further extending the SpaceTree approach by integrating navigation, animation, zooming, searching, and browsing (Lee et al., 2004). To support the task of comparing two different taxonomic representations, DoubleTree has been proposed and includes the representation of two TaxonTrees (Parr et al., 2004).

### 2.3 Hierarchical Visualization

Visualization facilitates the visual inspection and understanding of a given data (Fu et al., 2013). Hierarchical data visualization focuses on representing a part-to-whole relationship. Most efficient hierarchical visualizations represent the hierarchical structure of the data and make efficient use of the pixel or screen space (McGuffin & Robert, 2010; Burch et al., 2011).

The classical node-link tree diagram has been mostly used to represent simple hierarchical structures. As aforementioned, SpaceTree leveraged this classical structure with zooming functionality to improve the user’s focus. However, because of its inefficient use of space, a node-link diagram is not preferred when large data are considered. The authors represented the total number of child nodes that do not fit in the screen by a triangular symbol under the corresponding parent node; its size encoding said total (Plaisant et al., 2002). Many other tree structures focus on the efficient use of space to represent hierarchical data. Space-efficient visualizations enable space packing and maximize the pixel space. Tree-maps, icicles, and sunburst charts are the most prevalent visualizations (Woodburn et al., 2019).

Tree-maps take advantage of the total provided display space by mapping the hierarchical data to a rectangular area (Johnson and Schneiderman, 1991). A number of studies examined their efficiency by comparing multiple visualizations to the tree-map (Woodburn et al., 2019; Barlow & Neville, 2001, García et al., 2014). Many difficulties were revealed: the hierarchical structures that result in nested rectangular areas may become imperceptible, the understanding of a given hierarchy (relationship among elements and among siblings), the interpretation accuracy is lower compared to sunburst charts and icicles. Moreover, when two nodes located at different levels in the tree have the same size, the size of their corresponding rectangular areas are different. The interpretation accuracy decreased and was ascribed to this issue. Icicles represent hierarchical groups or clusters with juxtaposed layers and encode their size (Woodburn et al., 2019). Frequently used in cluster analysis and classification, they enable the linking or tracing back through the cluster in which the objects are located (Kruskal & Landwehr, 1983). Sunburst diagrams are accepted as the radial equivalent of icicles are extensively used (Rodden, 2014; Höllt et al., 2017). They show each level in the hierarchy through a series of concentric rings. The circle in the center represents the root node; each is sliced and divided according to its hierarchical relationship to the parent slice. Evaluation studies have determined that, although icicles use space less efficiently than sunburst diagrams, the hierarchy is easier to perceive (Kruskal & Landwehr, 1983; Barlow & Neville, 2001; Woodburn et al., 2019).

## 3 Materials

To provide a context-aware phylogenetic tree given a set of species, this work requires two data sets. The first includes the species and their distances to the common ancestors to create the phylogenetic tree view. The second data set lists the entire taxonomy of the species to create the icicle view. The materials section details certain criteria and assumptions on which the input data relies and the four different use cases. The former are taken from the Genome Taxonomy DataBase (GTDB). The latter follow the scope of the GTDB and include the domain categories of Archaea and Bacteria.

### 3.1 Input Data Criteria

The Genome Taxonomy Database (GTDB) with the release tag 202 is chosen as the source for the input data. It provides an ongoing census of bacterial and archaeal diversity through a phylogenetically consistent, rank normalized, and complete genome-based taxonomy (Parks et al., 2022). Although the GTDB is a groundbreaking database, many assumptions and criteria are considered. We detail below three important aspects relevant to this work.

#### 3.1.1 Genomic Criteria

The genomic sequences included in GTDB are mostly extracted from the National Center for Biotechnology Information (NCBI). This genomic dataset includes 254,090 bacterial genomes and 4,316 archaeal genomes. In order to eliminate low-quality genomic sequences, seven criteria are considered (Parks et al., 2020; Parks et al., 2022): the completeness estimate (>50%), the contamination estimate (<10%), the quality score (>50), the minimum required amount of marker genes (>40%), the maximum allowed number of contigs (<1,000 contigs), the sequence length in base pairs or N50 with a minimum length of five kilobase pairs (5 kbp), and the maximum allowed number of ambiguous bases (100,000).

Although these seven criteria can be adjusted for the genomic sequences with high taxonomic or nomenclature importance, low-quality genomes may be preserved due to their significance although they fail one or more criteria. For example, the isolate genome *Ktedonobacter racemifer* has 11% contamination. Yet, it still passes the related criterion and is accepted as a legitimate representative of the class *Ktedonobacteria* in the phylum *Chloroflexota*.

#### 3.1.2 Reference Trees

The bacterial reference tree is based on the concatenated alignment of 120 bacterial proteins called bac120 marker genes. The available tree is calculated under the Whelan And Goldman (WAG) model (Whelan and Goldman, 2001). It encompasses the representative genomes for each bacterial species cluster (Parks et al., 2020; Parks et al., 2022). The corresponding file follows the standard filename: bac120_<r202>.tree. The archaeal reference tree is based on the concatenated alignment of 122 archaeal genes. The available tree is calculated under the Posterior Mean Site Frequency (PMSF) model (Wang et al., 2018). The corresponding file follows the standard filename: arc122_<r202>.tree.

The internal nodes and the leaf nodes of these phylogenetic trees are annotated using the GTDB and the genomic accession numbers, respectively (Parks et al., 2022). Each tree includes non-parametric bootstrap support values provided in the Newick format. In addition, it is important to note that each tree contains only one representative genome for each GTDB species group. In other words, not all defined species are considered when constructing a given reference tree.

#### 3.1.3 Species Cluster

Notable achievements of the GTDB include the creation of an *operational species definition* that allows automatic assignment of genomes to species. This makes it possible to create species clusters from the huge genomic datasets available. In this regard, whole-genome ANI is accepted as a robust technique commonly applied. It allows the assignment of genomic sequences to representative GTDB genomes. The specific methods and criteria used in this process are briefly explained.

First, the genomes are evaluated by considering the aforementioned genomic criteria. Low-quality genomes are removed. The representative genomes are detected and assigned to the named species. In principle, all named species also meet the genomic criteria. The selection of the representative genomes follows one of the six metadata criteria: the type strain genomes of the species, the assembled genomes of NCBI from type material, the NCBI reference or representative genomes, the genomes collected from the type strain of subspecies, the NCBI Metadata and the ANI result between the genomes, or the manual investigation. A noteworthy criterion is the type strain of the species. It is the most used criterion for the selection of representative genomes.

Second, the ANI and the Alignment Fraction (AF) are the two methods applied to delineate species clusters. AF represents the percentage of the shared orthologous regions between two genomes. The ANI delineation radius for each representative species is set to 95%. However, if two representative species have an ANI greater than 95%, the defined value is adjusted to up to 97% of the ANI. Species representatives with an ANI greater than 97% are considered synonymous. The non-representative genomes are subsequently assigned to the representative species genomes to form the species clusters. In this regard, if a non-representative genome is within the ANI delineation radius of the nearest representative genome and meets the 65% AF criterion, it is assigned to that representative genome.

Third and last, the remaining genomes that cannot be assigned to any named species are arranged into *de novo* clusters. To select representative genomes, a greedy clustering algorithm is used and high-quality genomes are chosen as representative. The remaining non-representative genomes are then assigned to representative genomes considering the 65% AF criterion as in the case of named species clusters.

### 3.2 Use Cases

To demonstrate the relevance and performance evaluation of CAPT, four use cases are created. Two cases are taken from each domain: Archaea and Bacteria. By default, CAPT is available for download with all use cases. Data selections are made on the basis of reference trees provided by the GTDB and are further refined by decreasing the number of species considered and their corresponding accession numbers. The use cases include these selections as subsets of the original data to make them tractable. From the Archaea domain, the first couple of use cases comprises the class Methanomicrobia and the phylum Hadarchaeota. From the Bacteria domain, the second comprises the family Actinomycetaceae and the family Schwanellacea. We briefly detail each use case below.

#### 3.2.1 Class Methanomicrobia

Methanomicrobia contains the species that belong to the methanogenic Archaea group. The energy metabolism of these microorganisms consists of consuming carbon dioxide and producing methane. Organisms from this class play an important role in the global carbon cycle (Thauer et al., 2008).

#### 3.2.2 Phylum Hadarcheota

Hadarcheaota is a small phylum that contains organisms from the subsurface ecosystem. The metabolism of these organisms allows them to succeed in such environments thanks to genes involved in the oxidation of carbon monoxide and dihydrogen, with a potential coupling to the reduction of nitrite to ammonia. Earlier named Hadesarchaea (Baker et al., 2016), the GDTB follows the later proposed name Hadarcheota (Chuvochina et al., 2019).

#### 3.2.3 Family Actinomycetaceae

Actinomycetaceae is a family of bacteria classified in the phylum Actinobacteriota (Actinobacteria), which contains organisms from terrestrial or aquatic environments. These include gram-positive bacteria, whose cell wall consists of a thick layer of peptidoglycan (Schaal et al., 2006; Barka et al., 2016).

#### 3.2.4 Family Schwanellacea

Schwanellaceae is a family of bacteria classified in the phylum Proteobacteria. They comprise gram-negative bacteria, whose cell wall consists of a thin layer of peptidoglycan. Some members of Geobacteriaceae and Schwanellaceae have been considered as part of microbial fuel cells. They include metal-reducing bacteria, some of which have special membrane-bound cytochromes that can transfer electrons (Foulkes, 2011; Soni, 2007).

## 4 Methods

To complement phylogenetic information with phylogeny-based taxonomy, we offer linking two interactive visualizations which compose two simultaneous views: the phylogenetic tree view and the taxonomic icicle view. The proposed Context-Aware Phylogenetic Trees (CAPT) is an interactive web tool that supports users in exploration- and validation-based tasks. This section follows the nested model of visualization as a scaffold to systematically think about the design space. It details the domain, data and task abstractions, workflow, visual and interaction idioms, algorithm, and sensitivity analysis (Munzner, 2009).

### 4.1 Domain Situation

The domain situation describes the context of a visualization and often includes domain-specific terms. Phylogenetic studies aim to provide a comprehensive understanding of species lineages. That is, to examine the evolution of species or the process of change over time, in which entities diverge from a pre-existing organism (common ancestor). Phylogenetic trees are the most widely used visualization technique for showing evolutionary relationships between species. In these tree structures, the temporal aspect is represented by the use of different branch lengths, where longer times correspond to longer branches. The increase in the amount of genomic data available has affected the practice of taxonomy. In this regard, taxonomic classification has begun to be recovered from phylogeny to ensure consistent categorization. Motivated by the important role that such taxonomy plays in analyzing the evolution of entities through hierarchies, it is essential to provide taxonomic information to phylogenetic trees. This phylogeny-based taxonomy not only contributes to the consistency of biological analyses performed in phylogenetics but also strengthens the hypotheses formulated during the study of the evolution of organisms as well as the categorization of newly emerged species.

### 4.2 Data Abstraction

The data abstraction refers to what is shown to the user. The abstract vocabulary avoids domain-specific terms and refers to a translation process. In this work, two data abstractions are considered for the two views: the phylogenetic tree and the taxonomic icicle.

The phylogenetic tree view relies on the input data (i.e., Newick format) and its associated metadata (e.g., assembly accession numbers, estimated evolutionary time). The data abstraction is a node-link diagram or tree. The leaf nodes of the diagram are represented by text labels for species. The internal nodes represent the common ancestors of the species. The branches represent the estimated evolutionary times for the species.

**FIGURE 1:**
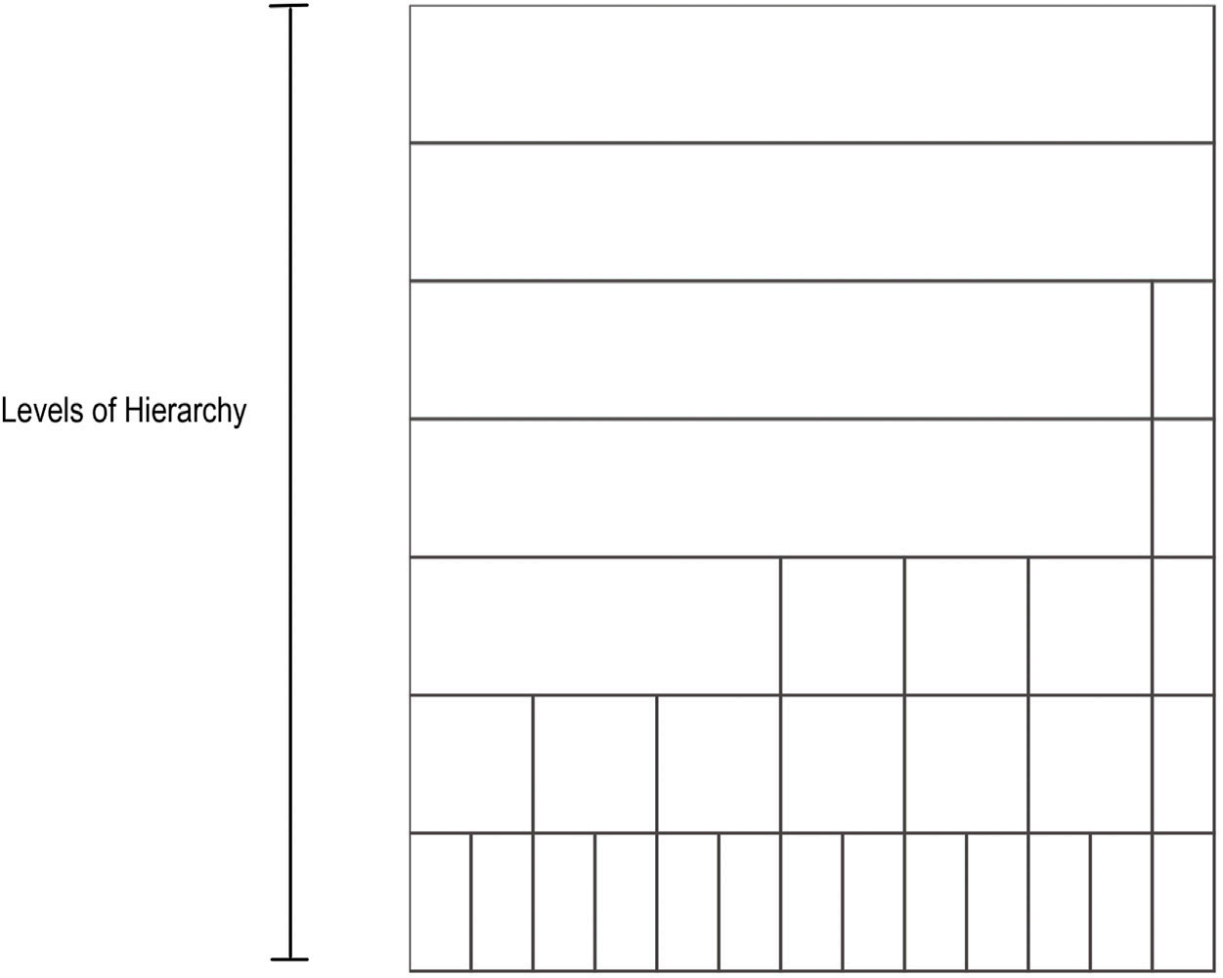
Example icicle visualization. A seven-level hierarchy or seven layers icicle is depicted. The number of layers and the partition shown in the layers varies from one icicle to another in accordance with the hierarchical and the categorical structure of the data.

The taxonomic icicle view is based on the main taxonomic ranks: domain, phylum, class, order, family, genus, and species. These ranks correspond to a qualitative variable in which the categories of the variable are not described by numbers but by verbal groupings. Since there is no order between the example categories in a rank, they follow the nominal scale. For example, the taxa Acidobacteria, Firmicutes, and Proteobacteria represent different unordered categories in the phylum rank. However, the defined taxonomic ranks follow a seven-level hierarchical structure. To generalize the domain problem, the taxonomic ranks can be considered as categorical nominal data belonging to the different levels of said hierarchy. Levels of the hierarchy are depicted as adjacent rectangular areas. The categories are located in the relevant area of the icicle view based on the level they belong to. Each area varies depending on the number of elements present in a given grouping. Figure 1 illustrates an example icicle visualization with a seven-level hierarchy or seven-layer icicle.

Compared to other visualizations, the icicle visualization is more efficient. For example, when nodes are located at different hierarchical levels, the corresponding rectangular areas of the different hierarchical ranks are not nested within each other, as is the case with a tree map visualization. In addition, the icicle allows size comparisons at each level of a given hierarchy, as opposed to a node-link diagram. Moreover, evaluation studies have shown that while icicle use space less efficiently than sunburst diagrams, the hierarchy is easier to perceive.

### 4.3 Task Abstraction

The task abstraction refers to why the user is looking at the visualization. To support the user in their tasks, different action and target pairs are made possible for both views. The actions include analyzing (consume, produce), searching (lookup, browse, locate, explore), and querying (identify, compare).

The phylogenetic tree view makes use of the analyzing action to examine how long species took to evolve through time and discover from which common ancestor they did. Presenting a phylogenetic tree with selection functionalities, including a Region Of Interest or an ROI, permits users to derive the taxonomic context for one or more selected species.

The taxonomic icicle view makes use of analyzing, searching, and querying. By default, a taxonomic icicle view is created for all the species in a phylogenetic tree view. The user may examine the resulting icicle visualization to discover the area size occupied by different taxa. By relying on the mouse click event, users can search the taxonomic path of a selected taxon. Identifying a taxonomic path of interest is task-dependent. Moreover, since it is possible to download each resulting icicle, users can compare the taxonomic rankings of different taxa or even species.

The link and brush functionality binds both views (c.f., 4.5. Visual and Interaction idioms). This supports searching actions to lookup (taxonomic rank and species known) and locate (taxonomic rank unknown but species known) across both views.

### 4.4 Workflow

CAPT follows the rule of thirds to display the two views side-by-side. By default, thirty percent of the page width is reserved for the phylogenetic tree view, while the rest is allocated for the taxonomic icicle view.

**FIGURE 2:**
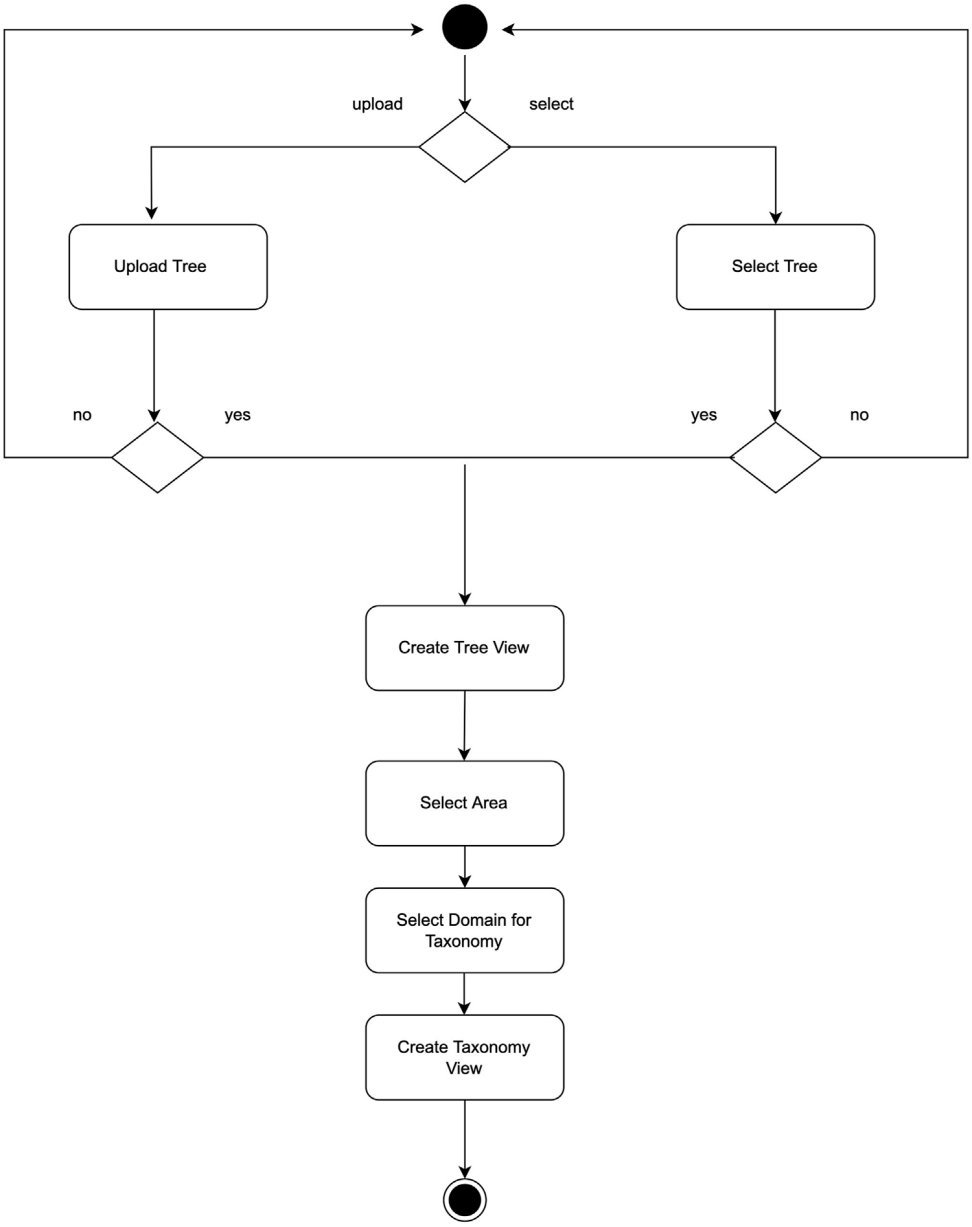
Workflow of the taxonomic icicle view creation. This workflow excludes the implemented interactions with the icicle visualization.

The user may start by either selecting data from the existing use cases, or uploading a tree file in the Newick format. In the latter case, this assumes that the assembly accession numbers are included in the uploaded tree. This enables automatic labeling of the leaf nodes. For instance, the GTDB relies on these accession numbers to label the phylogenetic reference trees and to differentiate the species in the taxonomy. Then, the user can proceed to select one or more species by using the click-and-drag event to draw a rectangular ROI. Once a selection is made, users may specify the domain of the taxonomy, and then a taxonomic view is created. The latter depends on a match between the selected species and a taxonomic domain. When a match is possible and falls under the GTDB taxonomy data of Archaea and Bacteria, the taxonomic icicle view is rendered. This workflow is visualized as a workflow diagram in Figure 2.

### 4.5 Visual and Interaction Idioms

#### 4.5.1 Phylogenetic Tree View

The phylogenetic tree view comprises three important parts: tree upload, tree, and area selections.

**FIGURE 3:**
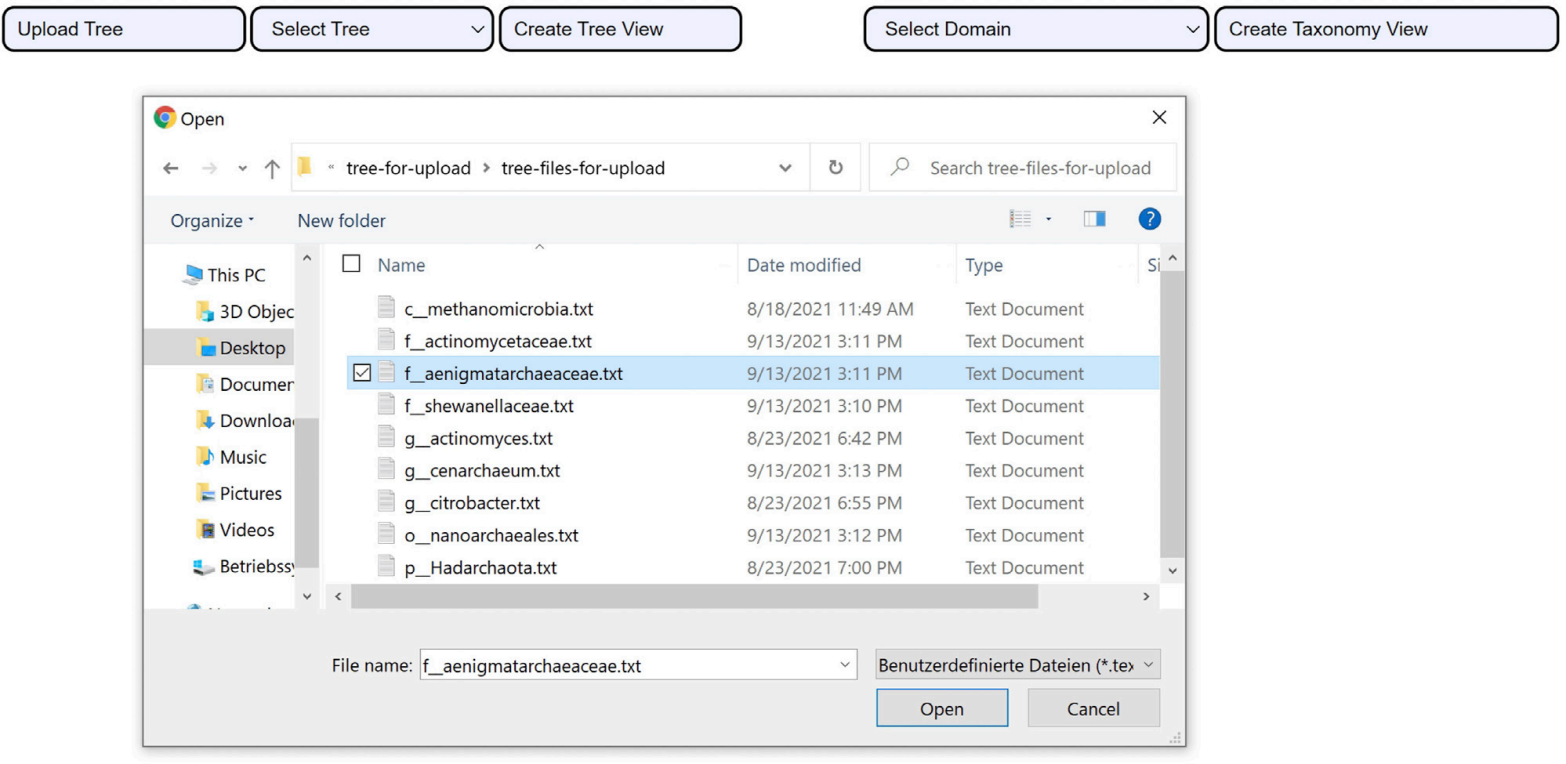
Uploading a tree using CAPT. After the file upload is successful. The user clicks on the *Create Tree View* button to visualize the uploaded phylogenetic tree.

The tree upload consists of uploading a tree file that meets three criteria: 1. the structure of the tree has to be encoded using the Newick format, 2. the species have to be labeled with the assembly accession numbers that the GTDB uses rather than their names, and 3. the file has to follow the UTF-8 text file format encoding. Once the file upload action is complete, the user can create the phylogenetic tree view as seen in Figure 3.

**FIGURE 4:**
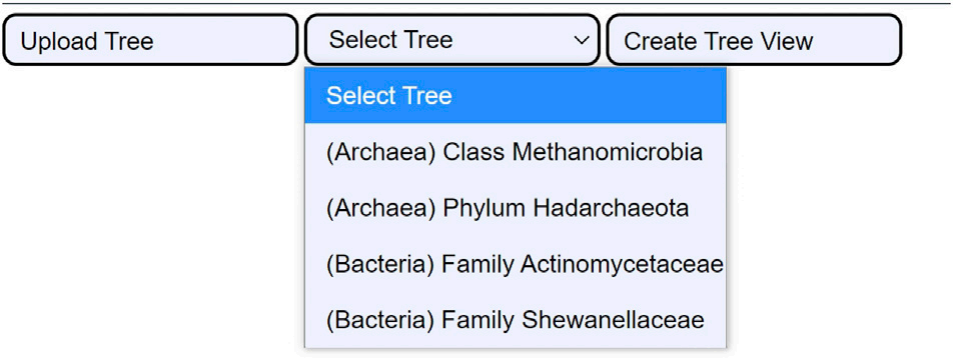
The four integrated use cases. When the user clicks the *Select Tree* button, a dropdown menu shows all four use cases. This permits loading the cached data for the four tree samples: the Class Methanomicrobia, the Phylum Hadarchaeota, the Family Actinomycetaceae, and the Family Shewanellaceae.

The tree selection consists of selecting one of the four integrated use cases (c.f., 3.2. Use cases). They are extracted from the reference trees of the GTDB. The use cases are ready examples that enable users to examine the utility of CAPT for exploration- and validation-based tasks. Their integration in the user interface is displayed in Figure 4.

**FIGURE 5:**
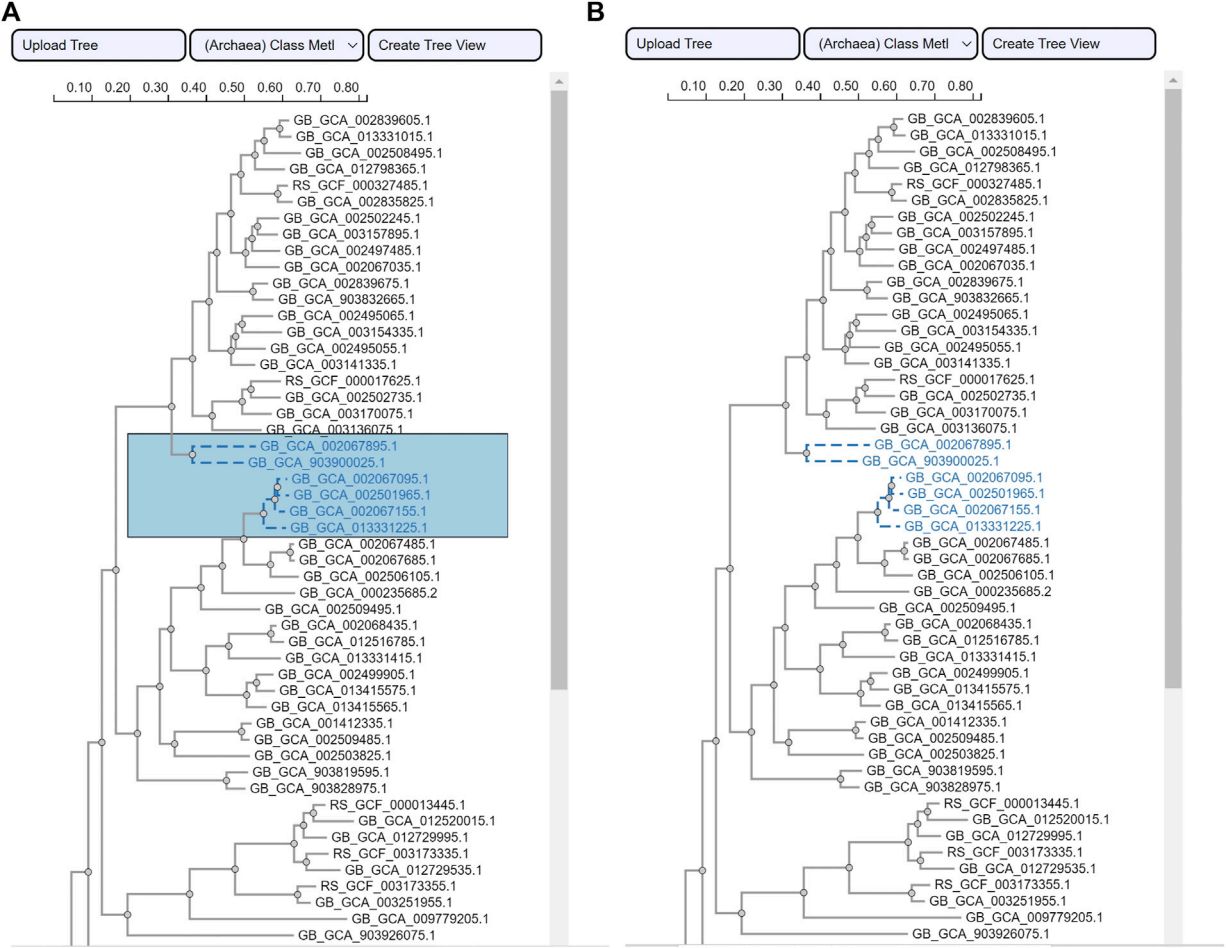
ROI selection for species using the phylogenetic tree view. Side-by-side screenshots of the phylogenetic tree view **(A)** while a user is selecting an ROI and **(B)** after the selection has been completed. The example tree is from the use case class Methanomicrobia.

The area selection provides users with a click-and-drag interaction to draw rectangular shapes over the phylogenetic tree view. It relies on the phylotree JavaScript package (Shank et al., 2018). To obtain the selected species in a ROI, an algorithm is implemented to retrieve the assembly accession numbers. To ensure visual consistency, while the click-and-drag interaction is active the color of the rectangular area is populated to the selected species once the selection is made (i.e., the user releases the mouse click). Figure 5 illustrates this interaction and visual encoding. The tree type is a rectangular phylogenetic tree.

The resulting assembly accession numbers are then used to retrieve the species taxonomy and create a taxonomic icicle view.

#### 4.5.2 Taxonomic Icicle View

The taxonomic icicle view comprises seven important parts: domain selection, partition, label, color, the tool-tip on mouseover event, the mouse click event in the icicle, and the export of an icicle visualization.

The domain selection is selected using the *Select Domain* button from a dropdown menu with two choices: Archaea or Bacteria. The chosen domain name has to correspond to the domain of the examined species within the selected phylogenetic tree view. Upon selecting the wrong domain, a warning popup box is displayed to inform the user. If a domain is incorrectly selected, the user’s selection in the phylogenetic tree is retained without the need to start over.

To create an icicle visualization, the partition layout is applied to the defined size of a Scalable Vector Graphics or SVG file format. The hierarchical layout relies on the D3 JavaScript package (Bostock et al., 2011). It creates the space-filling version of a node-link tree diagram, then the root of the grouped data is passed as a root to the partition layout, forming the nodes of the diagram. The partition layout initializes the nodes by considering the parent-child relationships in a given hierarchy. It returns the attributes of each node, including the two-dimensional coordinates; permitting the drawing of a custom area. The rectangles are appended to the SVG by relying on the node coordinates. To improve the readability of the lengthy species labels, all rectangles drawn at the lowest layer of the species rank are adjusted to their minimum size.

Each rectangular area of the icicle visualization is labeled with its corresponding taxa name. The font and font size are inherited from the phylogenetic tree for a consistent display across views. To increase readability, the species labels are displayed diagonally or rotated at a 45-degree angle; outside the rectangular areas. By following the nomenclature rule, species names include their genus taxon name. Due to the lengthy textual information, the labels of family and genus ranks are displayed vertically or rotated at a 90-degree angle. All other labels are displayed horizontally.

The color encodings are chosen based on the seven taxonomic ranks. Hence, the icicle visualization has seven layers. Due to the hierarchical nature of the data, a sequential color palette is preferred. To create it, we relied on the ten simple rules to colorize biological data visualization (Hattab et al., 2020). The blue hue is considered. A threshold is used to clip the lower and higher values of the blue sequential color palette. To achieve maximum contrast for the species, the white color is added manually for the lowest layer. The resulting color palette is applied by using the depth attribute. Table 1 reports the color palette. The orange complementary color of the blue (domain) is selected to encode tool-tip interaction, as described below. While the blue/orange colors are colorblind-friendly, the seven-layer icicle visualization is not suitable for photocopying.

**TABLE 1:** Color encodings in the taxonomic icicle view. The seven different levels or layers of the icicle are reported with their HEX color codes and a render of the employed sequential color palette. The last color in the color palette corresponds to white and is represented by a white square.

Levels	HEX color code	Color palette
Domain	#2171B5	■
Phylum	#4292C6	■
Class	#6BAED6	■
Order	#9ECAE1	■
Family	#C6DBEF	■
Genus	#EFF3FF	■
Species	#FFFFFF	□

The tool-tip on the mouseover event introduces focus and clarity whenever the user requires further information. The tool-tip includes three attributes: the name, the id, and the size. The id refers to the species. Upon a mouseover event atop a label of a rectangle, the tool-tip is displayed. By default, the tool-tip is displayed on the left of a label. This guarantees that the tool-tip is never outside of the screen bounds if the mouse cursor is close to the screen edge (on the right). The examined species is colored in the complementary orange #B56521 of the blue #2171B5 (domain). To provide phylogenetic and taxonomic context for the examined species, a link and brush functionality connects both views using the color channel. The examined species is colored orange #B56521 simultaneously in the phylogenetic tree and in the taxonomic icicle view, respectively.

The mouse click event in the icicle visualization supports search actions (lookup, browse, locate, explore). Upon clicking on a rectangular area of the icicle, the icicle visualization is updated and a smaller icicle is rendered, showing the taxon of interest. The mouse click event filters the path of the selected taxon to include all the taxonomic ranks between the highest rank of the domain and the rank of the selection. To return to the initial icicle, before filtering, the user clicks on the same or any other rank of interest in the current icicle. Consistent color encodings are used throughout the icicle navigation; c. f., Table 1.

Exporting the icicle visualization is possible by clicking on the *Export* button. This saves an SVG file of the current icicle.

### 4.6 Algorithm

To provide the context to the phylogenetic tree or a selection comprising a subset of species, an algorithm is implemented to retrieve the taxonomy of the selected species from the associated taxonomic data. This relies on a matching function between the selected species in the phylogenetic tree view and the taxonomy data. The central part of this function is shown below; where one element corresponds to one species.


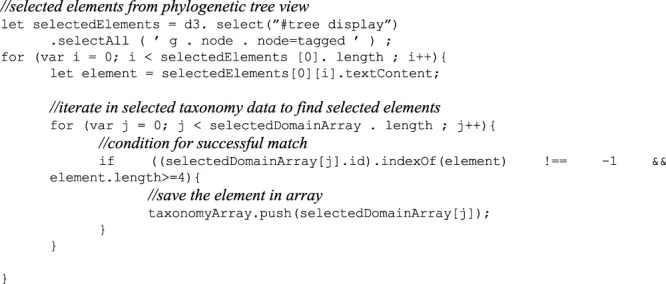


**FIGURE 6:**
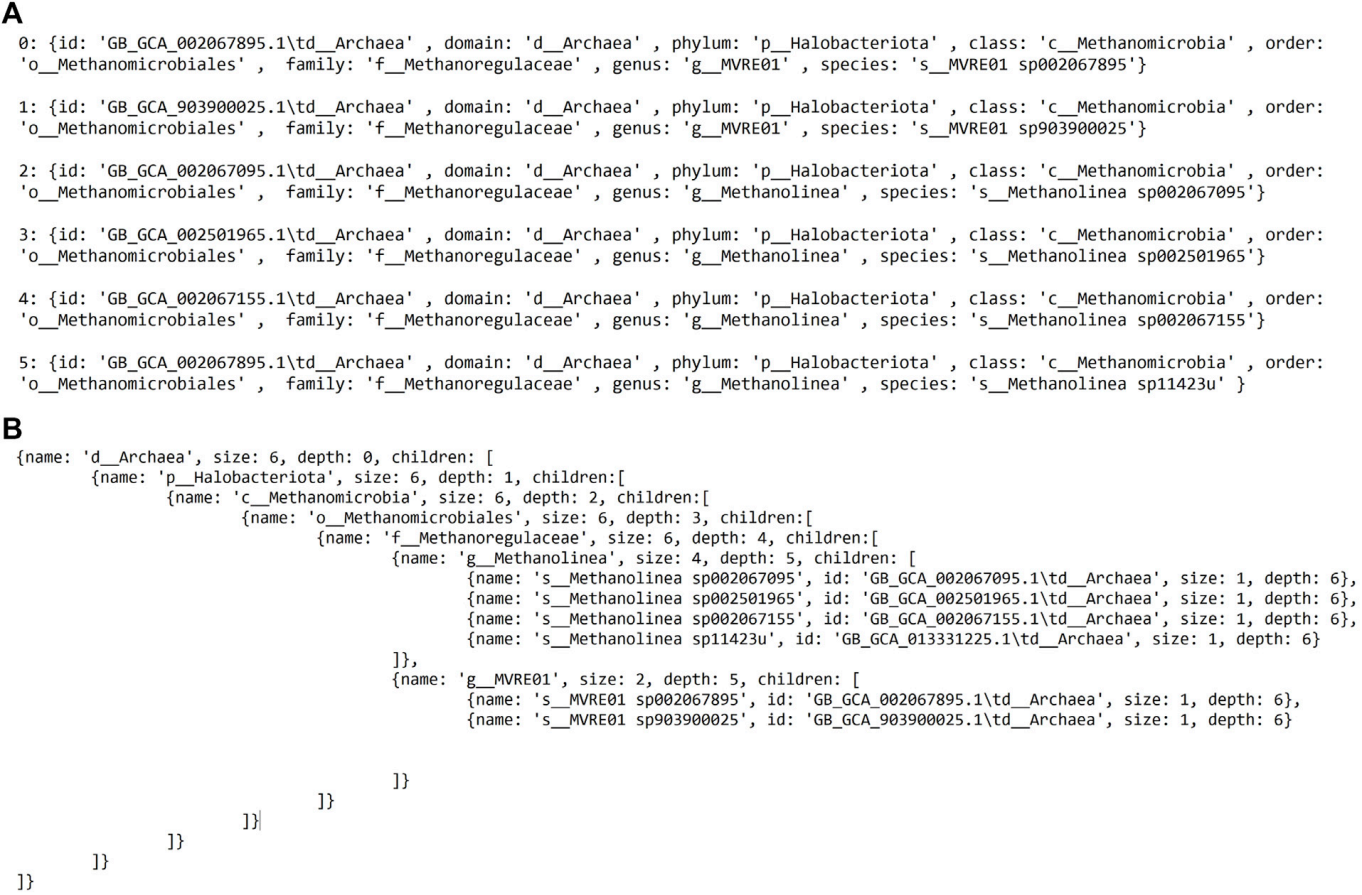
Before and after the algorithm is applied. **(A)** The structure of the data before. **(B)** After the algorithm successfully completes matching, grouping, and further attributes have been added.

Indeed, the number of iterations in the inner for loop is independent of the outer one. Given *n* and *m* the selected elements size length and the selected domain array size, the inner loop executes *m* times whenever the outer one performs an iteration. This results in an n*m in total. The time complexity to obtain the taxonomy data of *n* species from the phylogenetic tree view is *O* (n*m). Since the required space increases with respect to the array size with *n* elements, the space complexity is linear *O* (*n*). Upon a successful match, this returns the selected species with their seven taxa as separate JavaScript objects. The grouping starts at the species rank and iterates until all ranks have been visited, saving the grouping as a child to the rank above. Further attributes are added for each object: the size and the depth. A before and after example is provided in Figure 6.

### 4.7 Sensitivity Analysis

A sensitivity analysis is performed to evaluate the time required to draw the icicle visualization for a different number of selected species; five scenarios or data selections are created. Two experiments are considered. First, the data processing, including the algorithm and the drawing of the icicle visualization. Second, only the drawing of the visualization. For each experiment, the average elapsed time and the standard deviation are calculated for 1,000 runs. This totals 10,000 runs. To examine the computational complexity, regression models are fitted to the results. The data used for the sensitivity analysis is the use case from the Methanomicrobia class. Time measurement is performed using the Performance API^1^. The elapsed times are reported in milliseconds (ms). The Google Chrome version 94.0.4606.61 web browser is used.

## 5 Results

With the help of interactive techniques such as brushing and linking techniques in side-by-side views, our tool guarantees constant frame rates. CAPT is well suited for exploration and validation-based tasks in phylogeny-based taxonomy. The source code and the four use cases are available at https://github.com/ghattab/CAPT. Sample results for the use cases are given below. In addition, the results of the sensitivity analysis and the fitting of the regression model are presented in detail.

**FIGURE 7:**
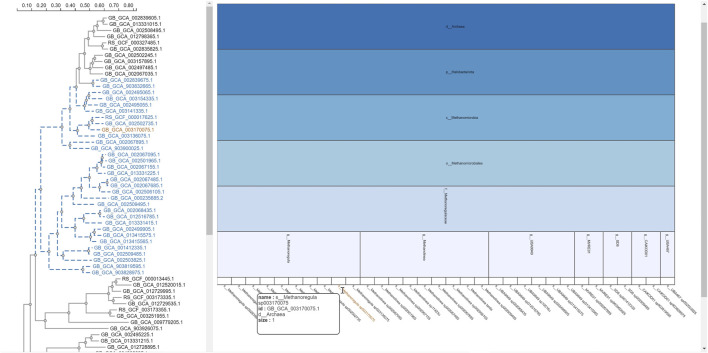
Taxonomic icicle view of a selection from the tree class Methanomicrobia. The mouseover event over the label of the species Methanoregula sp003170075.1 brings up a tool-tip which includes the species name, the species id: GB GCA 003170075.1 and the size of 1.

**FIGURE 8:**
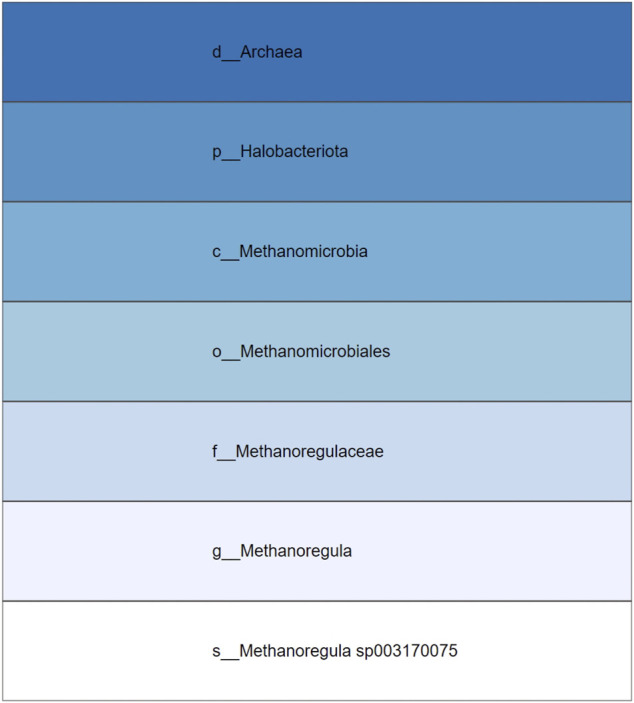
Icicle visualization of the Methanoregula sp003170075 species from the tree class Methanomicrobia.

**FIGURE 9:**
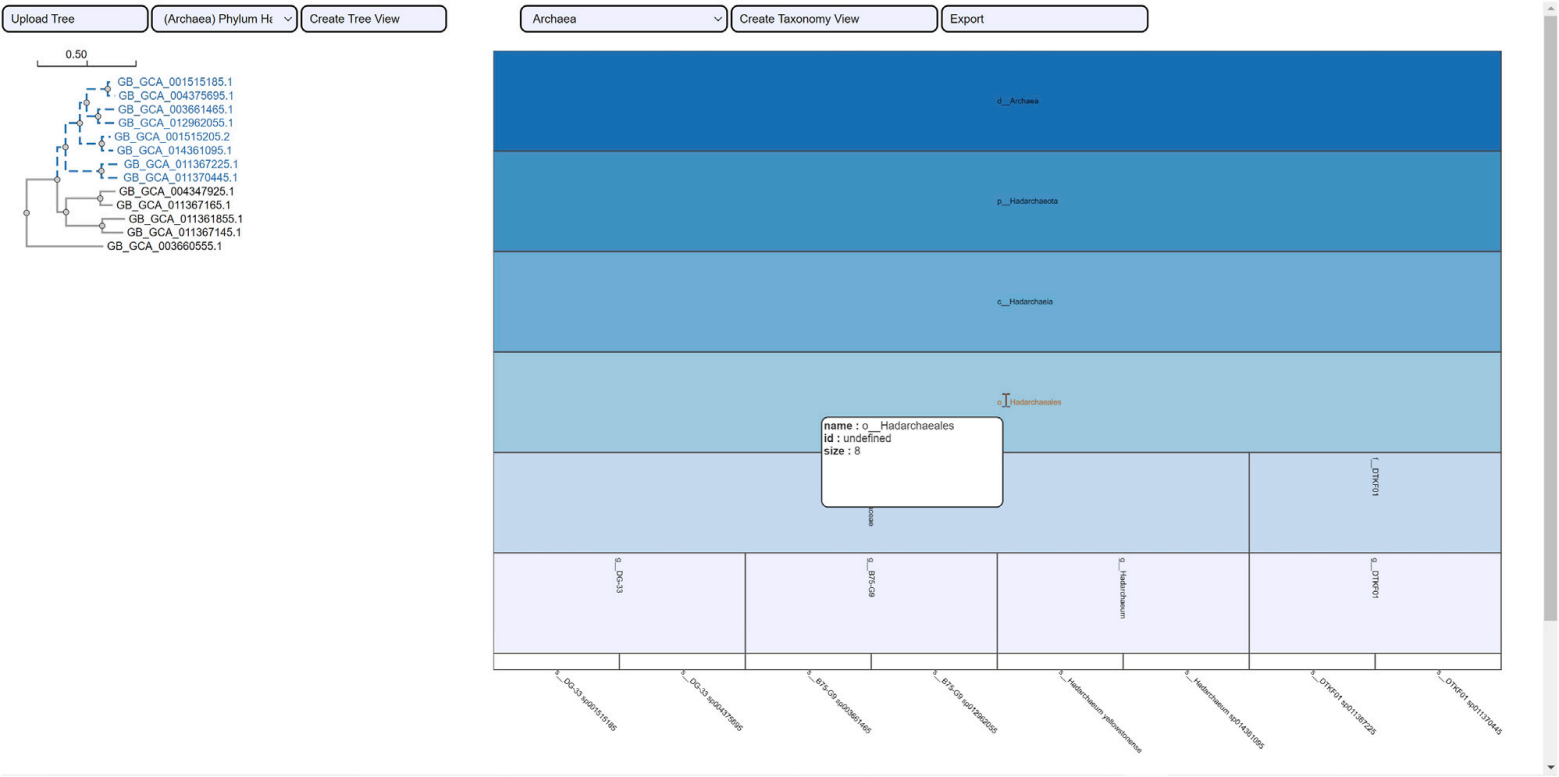
Taxonomic visualization of a selection from the tree phylum Hadarcheota. The mouseover event over the label of the order Hadarchaeales brings up a tool-tip which includes the order name, the id: undefined and the size of 8. Two families and four genera are observed within the phylogenetic ROI.

**FIGURE 10:**
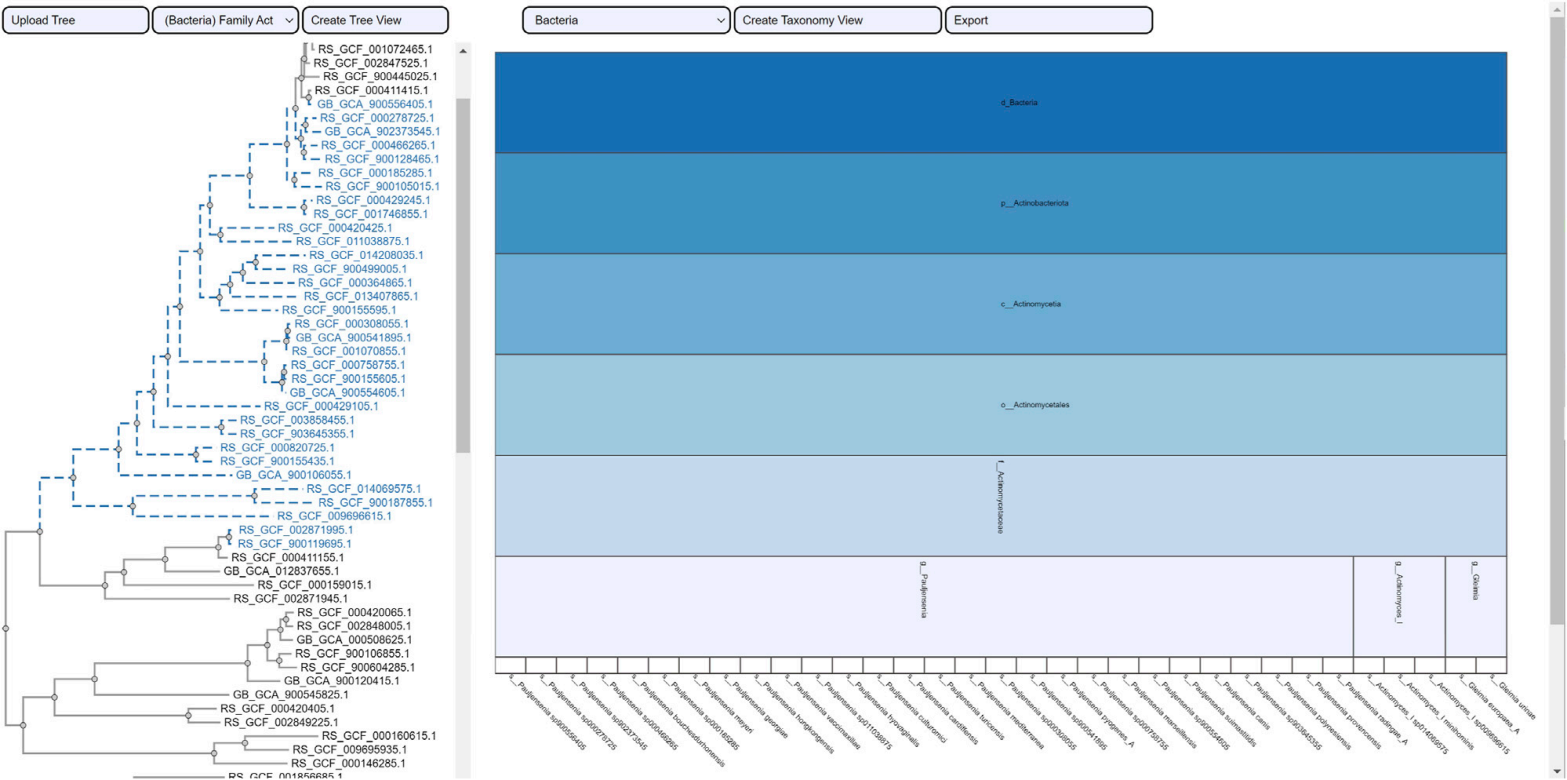
Taxonomic visualization of a selection from the tree family Actinomicetaceae. Three genera are observed within the phylogenetic ROI.

**FIGURE 11:**
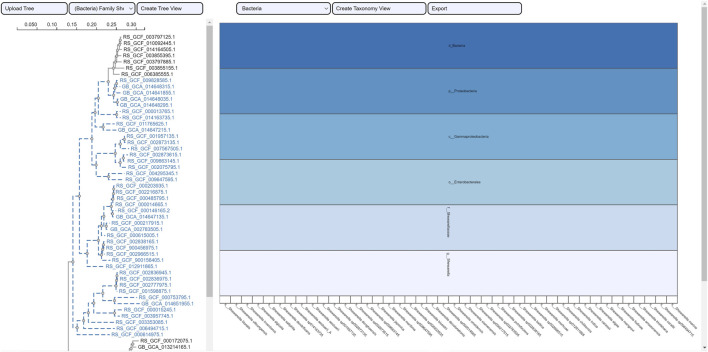
Taxonomic visualization of a selection from the tree family Schwanellacea. The resulting icicle visualization shows a hierarchy with a common taxonomic classification for all the reported species.

The selection of an area in a phylogenetic tree view and the subsequent creation of the taxonomic icicle view was depicted using the class Methanomicrobia in Figure 7. The mouseover event highlighted the species across both views by changing the font color to orange and was useful for large data. Upon clicking on the highlighted species Methanoregula sp003170075, the taxonomic icicle view can be refined as seen in Figure 8. This interactive feature permitted us to go back and forth to look up a species, browse a taxon, locate certain species, explore a phylum. Each of the remaining three use cases followed an ROI-based selection to depict the resulting phylogenetic and taxonomic views: Figures 9–11 for the trees of the phylum Hadarcheota, the family Actinomycetaceae and the family Schwanellacea, respectively. Figures 9, 10 showed varying structures in the hierarchy levels. To further explore these use cases, they are by default made available with the CAPT web tool at the following address: https://capt.mathematik.uni-marburg.de/.

CAPT was benchmarked by evaluating the time required to draw the icicle visualization without and with the implemented algorithm. The sensitivity analysis investigated the time required to draw the icicle visualization for different numbers of species in an ROI. Tables 2 and 3 show the results obtained excluding or including the algorithm, respectively. Each row reports the average elapsed time and the standard deviation for 1,000 runs. Results have shown that including the algorithm increased the elapsed time on average by 2.89 fold. As observed in the tabular data, the rendering of the icicle visualization followed a linear trend. This underlined the fact that the number of elements in the taxonomic data plays an important role.

**TABLE 2:** Sensitivity analysis to draw the icicle visualization. Values are reported in milliseconds (ms).

Number of selected species in ROI on phylogenetic tree	Average elapsed time (ms)	Standard deviation (ms)
20	2.92	2.61
40	4.62	6.09
60	7.11	7.24
80	9.05	8.37
100	11.04	9.75

**TABLE 3:** Sensitivity analysis to draw the icicle visualization and run the algorithm. Values are reported in milliseconds (ms).

Number of selected species in ROI on phylogenetic tree	Average elapsed time (ms)	Standard deviation (ms)
20	7.41	2.31
40	14.55	6.84
60	20.56	8.81
80	26.96	9.59
100	31.61	13.24

**FIGURE 12:**
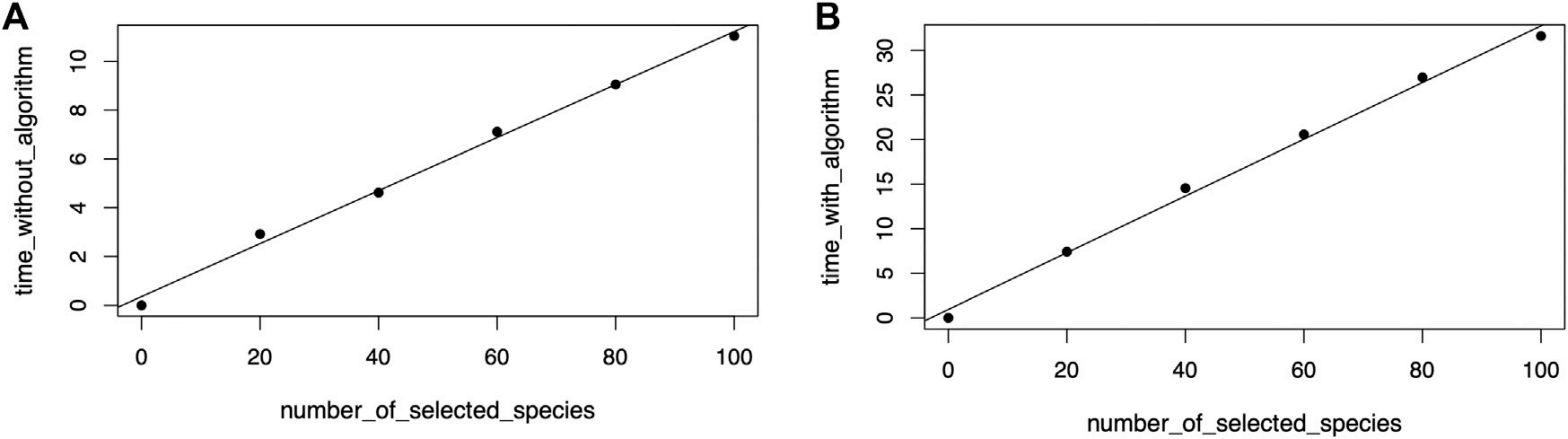
Linear model fitting for the sensitivity analysis to draw the icicle visualization **(A)** and including running the algorithm **(B)**.

Regression model fitting confirmed the linear relationship between the number of selected nodes or species in the phylogenetic tree view and the time required to draw the icicle visualization in the taxonomic icicle view. Figure 12A shows the resulting linear model with a correlation value of 0.99. When the algorithm was included in the benchmark, the model fitting also showed a linear relationship with a correlation value of 0.99. The calculated data points are plotted in Figure 12B.

## 6 Discussion

Several points are worth discussing. As technological advances push the boundaries of what is possible, future demand aligns with more integrative tools that facilitate multiple views, provide context, and dynamically link technological and conceptual aspects. Indeed, the influence of the increasing amount of genomic data on the practice of biological taxonomy is evident. Although CAPT introduces a practical solution while working in phylogeny-based taxonomy, certain points require further consideration.

Future versions of the tool should include certain interaction techniques that enable view-specific enrichment of the phylogenetic tree view. For example, the addition of phylogeny-specific metrics such as the bootstrap value. Moreover, Gadagkar et al., 2005 propose, in addition to the bootstrap support, to report the number of individual genes supporting an inferred clade in the concatenated sequence tree.

Species names are problematic and a source of continuous discussion in the world of taxonomy. The authors preferred to rely on the genomic accession numbers as they constitute a reliable data attribute. Although more reliable, they constitute a problem for easy interpretability and correspondence to species names. Conceptually, this requires the user to define a focus area to differentiate the represented species in the phylogenetic tree view from the accession numbers. The combination of the ROI and the tool-tip functionalities helps alleviate this problem and decrease the conceptual distance between phylogeny and taxonomy.

The provided use cases served as a starting point. Many studies could have benefited from using our tool to support exploration or validation tasks in various contexts. For example, Hailu et al., 2021 focused on the methanogenic stage of microbial communities, i.e., microbes responsible for the biomethanation of the substrates. Related to the class Methanomicrobia use case and out of the methanogens genera, they found that Methanosa and Methanosarcina were the most dominant in this biochemical activity. They are distinguished by the type of metabolite they depend on. Indeed, both genera fall within the same order known as Methanosarcinales and mainly use acetate, while the Methanobacterium belongs to the Methanobacteriales order and mainly uses dihydrogen (Hailu et al., 2021). Further examples could provide important context and support domain experts in their tasks. For example, the phylogeny-based delimitation of species boundaries.

CAPT presupposes phylogenetic tree data with explicit species names and their genomic accession numbers. Indeed, the process of amending a phylogenetic tree with these accession numbers can prove time-consuming. Given a large phylogenetic tree, further efforts are required to develop semi- or fully-automated enrichment approaches.

Although various interactive functionalities are implemented for the taxonomic icicle view, further improvements are possible such as fitting the scale of the icicle visualization. Considering user-settable zoom-in functionalities may improve readability and user-based customization. This includes a reset button to revert to a default scale. To parallel the addition of the bootstrap value and the number of individual genes supporting an inferred clade in a phylogenetic tree view, further details may be added in the taxonomic icicle view. For instance, in the tool-tip, the exact ANI and AF results of each species’ genomic sequence may be reported.

The icicle visualization is an efficient solution as it enables size comparisons at each level of a given hierarchy. However, when very large phylogenetic tree data is imported, and their taxonomic icicle view is created; an under-resolved icicle visualization results. Indeed, an increase in the number of selected species decreases the number of pixels allocated for the rectangular areas in the icicle at the species layer. The aforementioned zoom-in functionality could prove useful with the currently implemented SVG file format. Moreover, with large phylogenetic trees, labeling the icicle visualization also becomes problematic. This is because the names of the taxa are often quite long. Future possible improvements may involve user-defined regular expressions to simplify a label set.

CAPT meets many technological and design requirements. It facilitates the creation and visualization of a phylogeny-based taxonomy and provides taxonomic context. Moreover, CAPT could be used to visually compare the quality of different taxonomies made available by different databases for one phylogeny of interest. Overall, this work provides a tool with state-of-the-art performance and a useful set of functionalities for phylogeny-based taxonomy visualization.

## Data Availability

The original contributions presented in the study are included in the article/Supplementary Material, further inquiries can be directed to the corresponding author.

## Footnotes

1
https://developer.mozilla.org/en-US/docs/Web/API/Performance.
